# Prevalence of *Toxoplasma gondii, Leptospira* spp., and *Coxiella burnetii-*associated antibodies in dairy cattle with reproductive disorders

**DOI:** 10.14202/vetworld.2022.2844-2849

**Published:** 2022-12-15

**Authors:** V. Balamurugan, K. Vinod Kumar, Anusha Alamuri, P. P. Sengupta, G. Govindaraj, B. R. Shome

**Affiliations:** Indian Council of Agricultural Research-National Institute of Veterinary Epidemiology and Disease Informatics, Bengaluru, Karnataka, India

**Keywords:** dairy cattle, leptospirosis, Q fever, seroprevalence, toxoplasmosis

## Abstract

**Background and Aim:**

In cattle dairy farms, abortions and other reproductive problems due to major infectious diseases are overlooked, and identifying their causative agents is very challenging without a confirmatory diagnosis. Further, a prevalence study in animals will provide important hints of pathogen reservoirs and provide necessary direction to disease burden with appropriate control and biosecurity measures at the farm level. This study aimed to estimate the prevalence of *Toxoplasma gondii* antibodies in dairy cattle associated with reproductive problems along with coexisting antibodies against abortifacient zoonotic (*Coxiella burnetii* and *Leptospira* spp.) pathogens.

**Materials and Methods:**

Cattle sera (n = 246) from dairy farms (n = 35) situated in different locations in India were screened for anti-*T. gondii* and *C. burnetii* antibodies with enzyme-linked immunoassay and *Leptospira* spp. antibodies with microscopic agglutination test.

**Results:**

The overall prevalence of 11.4% (95% confidence intervals [CIs]: 7.99%–15.96%) antibodies in cattle associated with reproductive problems (p < 0.021) with farm-level seropositivity of 43% was observed. Further, on analysis of screened sera, 49.8% (95% CI: 42.6%–55%) and 77.6% (95% CI: 72%–82.4%) of samples were found to be positive for *C. burnetii* and *Leptospira* spp. antibodies, respectively. Moreover, the seropositivity of 91.9% (226/246) for at least one of the screened zoonotic pathogens was observed, indicating antibodies against either of these organisms in association with reproductive disorders (p < 0.005). The percentage of cattle found to have *T. gondii* antibodies was only 1.8%, whereas 11.5% and 41.6% of cattle were found to have *C. burnetii* and *Leptospira* spp. antibodies, respectively. Nevertheless, the predominantly mixed infections observed were of *Leptospira* and *C. burnetii* (34.5%), followed by all three infections (4.9%); toxoplasmosis and leptospirosis (3.5%); and toxoplasmosis and Q fever (2.2%).

**Conclusion:**

The serological detection of antibodies against these pathogens in cattle may have significant implications for the livestock industry and public health, suggesting the need for continuous surveillance and monitoring of these infections to prevent their spread.

## Introduction

Toxoplasmosis is one of the significant zoonotic diseases of public health importance caused by *Toxoplasma gondii*, an obligate and intracellular sporozoan parasite. The disease is distributed worldwide with varied frequency in different regions and is most common in areas with warm, moist climates, and low-lying areas [1]. *Toxoplasma gondii* is widely prevalent and can infect almost all warm-blooded species as intermediate hosts, including humans. However, felid/domestic cats are the only known definitive hosts where the organism may undergo sexual reproduction. These hosts are responsible for spreading infection through fecal contamination of pastures, food, and water, with oocysts causing severe diseases in animals and humans. Humans can get the infection by ingestion of raw/inadequately cooked meat (risk factors) contaminated with parasite cysts (tachyzoites/bradyzoites) of *T. gondii* [2] or consumption of contaminated food or water with oocysts or transplacental transmission in humans and the predators through carnivorism [1, 3, 4]. The infection causes significant economic loss (USD 5–15 million for the Great Britain and Uruguay) due to abortions, reproductive disorders, and other developmental disabilities in livestock and humans [1]. Bovines can be infected either transplacentally or by ingesting feed or water contaminated with the oocysts shed by a definitive host. Infected animals develop cysts in their tissues that contain the bradyzoites of parasites and are the primary source of infection. *Toxoplasma gondii* infection is usually subclinical in livestock and is the common and significant cause of the reproductive problems in sheep and goats, whereas, in cattle, perinatal mortality or abortion has not been recorded [5]. Toxoplasmosis is uncommon in cattle; however, symptoms of pyrexia, dyspnea, nervous signs, lethargy, and sometimes stillborn calves and neonatal deaths may be seen if the disease occurs in adults.

The incidence of toxoplasmosis in cattle is not high, but seropositivity is high in different topographical and environmental conditions. Although several studies were conducted, the information on the seroprevalence of toxoplasmosis in cattle is limited. Data on the prevalence of toxoplasmosis in domestic animals and their potential risk as reservoirs from India are scanty, though a few reports exist [6, 7]. Many neglected zoonoses occur mainly among occupational risk group personnel (animal handlers, veterinarians, butchers, and dairy workers). Over the years, livestock owners in several regions have reported unusual abortions and other reproductive problems in cattle that remain undiagnosed. The parasite *T. gondii* and the bacteria *Coxiella burnetii* and *Leptospira* spp. are well-established abortifacient zoonotic pathogens of public health importance and have a wide range of hosts that manifest themselves in several forms, including the reproductive disorders in bovines [8–10]. In dairy farms, abortions and other reproductive problems due to brucellosis, leptospirosis, trichomoniasis, Q fever, etc., are overlooked, and identifying their causative agents is very challenging without confirmative diagnostics. Further, the etiological factors of these diseases that affect health/production are still poorly known. Besides toxoplasmosis, leptospirosis, and coxiellosis/Q fever, infectious zoonotic diseases affect humans and domestic and wild animals. The studies on the prevalence of these infections in dairy cattle in various regions of India have shown that Q fever and leptospirosis are an important menace to public health [11, 12] and in livestock by causing abortions and reproductive problems with the reduction in their milk production and febrile illness in humans.

Serosurveys on prevalence of these diseases in animals will provide important hints of pathogen reservoirs and provide necessary direction for its mitigation by employing appropriate control and biosecurity measures at the farm level. Further, the studies on the prevalence of toxoplasmosis in cattle provide the disease status *per se* and are imperative for developing preventive measures in humans. In India, a limited number of reports on cattle toxoplasmosis are available [6, 7, 13]. Nevertheless, its diagnosis is challenging and infection was investigated by various serological screening assays [14]. Hence, this study aimed to estimate the seroprevalence of toxoplasmosis in cattle to establish the status of anti-*T. gondii* antibodies associated with reproductive problems in the various dairy cattle farms located in different geographical areas of India. In addition, the relative importance of the cattle reproductive disorders and their coexisting antibodies or exposure to the selected abortifacients pathogens (*T. gondii*, *C. burnetii*, and *Leptospira* spp.) of the cattle were investigated.

## Materials and Methods

### Ethical approval

The manuscript does not contain an experimental animal trial. This work was carried out in the Indian Council of Agricultural Research-National Institute of Veterinary Epidemiology and Disease Informatics (ICAR-NIVEDI) in the Institutional Biosafety Committee approved Project (F. No. 6–52/NIVEDI/Biosafety/2016/07–19). No ethical clearance is required for collecting small volumes of blood samples required for seroepidemiological studies, as per Committee for the Purpose of Control and Supervision of Experiments on Animals guidelines of India. Moreover, these serum samples were collected by well-trained professionals (veterinarians) concerning animal welfare regulations and submitted to the ICAR-NIVEDI for disease diagnosis.

### Study period and location

The study was conducted from April 2015 to March 2021. Cattle sera from dairy farms situated in different states (Haryana, Jharkhand, Sikkim, Madhya Pradesh, Chhattisgarh, Gujarat, Uttarakhand states; Andhra Pradesh, Karnataka, Tamil Nadu, Telangana, and Maharashtra) of India available in the Leptospirosis Research Laboratory of ICAR-NIVEDI were employed in this study. The samples were processed at Leptospirosis Research Laboratory of ICAR-NIVEDI.

### Sample size and sera

The sample size of 246 was determined as per formula N = Z^2^ [p (1-p/e^2^] {where, N = sample size, Z = 95% confidence level, p = 20% expected proportion {prevalence was considered based on the published reports in cattle with reproductive disorders [6, 15, 16], and e is the precision level (5%)} using Epitools (http://epitools.ausvet.com.au/content.php?page=1Proportion) for the prevalence study of *T. gondii* antibodies in dairy cattle. The serum samples from dairy cattle associated with a history of reproductive problems (repeat breeding, infertility, anestrus, abortions, endometritis, etc.) were collected from dairy farms (n = 35) located in different geographical areas. The details of the serum samples screened from different farms located in various states of India are summarized in Table-1. In general, *T. gondii* infection is often highest in the niche that has hot, humid climates, and lower altitudes, because the oocysts survive better in these type of environments. The serum samples were either collected by the authors’ teams or submitted by the field veterinarians as suspected samples to the ICAR-NIVEDI for diagnosis of diseases. The collected sera were transported on the ice to the laboratory for testing and stored at −20°C until further use.

**Table-1:** Details of the tested sera from cattle for antibodies against *Toxoplasma gondii*, *Coxiella burnetii,* and *Leptospira* spp*.* and their results.

Region	No. of farms screened	Total no. of samples screened	No. of farms positive for			No. of serum samples positive for			Percent positivity of samples for		
		
			Toxoplasmosis	Q fever	Leptospirosis	Toxoplasmosis	Q fever	Leptospirosis	Toxoplasmosis	Q fever	Leptospirosis
North	15	103	6	15	14	13	37	82	12.62	35.92	79.61
South	20	143	9	18	20	15	83	109	10.49	58.04	76.22
Grand total	35	246	15	33	34	28	120	191	11.38	48.78	77.64
						95% confidence intervals			(7.99–16)	(42.6–55)	(72–82.4)

Chi-square value (χ^2^) = 5.21** (toxoplasmosis); 10.21***(Q fever); 5.52** (leptospirosis); χ^2^=10.25 ***(toxoplasmosis or Q fever or leptospirosis) **and ***indicate 5% and 1% levels of significance, respectively. North region comprising sampled farms from Haryana, Jharkhand, Sikkim, Madhya Pradesh, Chhattisgarh, Gujarat, and Uttarakhand states; South region comprising sampled farms from Andhra Pradesh, Karnataka, Tamil Nadu, Telangana, and Maharashtra states

### Serology

#### Toxoplasmosis

The serum samples were screened for *T. gondii* antibodies using *T. gondii* ruminant kit (Bovine *T. gondii* Antibody GENLISA™ enzyme-linked immunoassay [ELISA] [Krishgen Biosystems, India Cat. No: KLB30038]). Briefly, the test sera were diluted to 1:100 in sample diluent and 100 μL was used along with controls. Then, the plate was incubated at 37°C for 30 min. After incubation, the wells were washed 4 times with 250 μL wash buffer per well. Further, 50 μL of horseradish peroxidase conjugate was added to each well, and after 30 min of incubation at 37°C, the wells were washed. Then, 100 μL of tetramethylbenzidine substrate was added to each well and incubated at room temperature (25–28°C) for 10 min in dark conditions. After incubation, 50 μL of stop solution was added and the plate was read at wavelengths of 450 nm with 620 nm as a background reference on a microplate reader. The optical density (OD) value >0.25 plus absorbance of the negative control mean is considered positive, whereas the OD value lesser than 0.25 plus absorbance of the negative control mean is considered negative. A farm was considered positive for toxoplasmosis when at least one animal was found to be positive for antibodies on detection.

#### Q fever

The sera were also subjected to the detection of *C. burnetii* antibodies using PrioCHECK™ Ruminant Q Fever AB Plate ELISA Kit (Applied Biosystems, Thermo Fisher Scientific, USA), and the performance of the assay and interpretation of results were carried out as described earlier [12]. Briefly, the 100 μL of diluted serum samples (1:400 in B1 sample buffer) were added into the pre-coated plate along with control panels as per the given format. Then, the plate was incubated at 37°C for 1 h, and after incubation, the plates were washed three times with 300 μL of the wash buffer/well. Then, 100 μL of conjugate/well was added, and after 1 h of incubation at 37°C, the wells were washed three times. Further, 100 μL of substrate solution/well was added and incubated for 10 min in the dark at 25–28°C Then, 100 μL of stop solution/well was added and the plate was read at wavelengths of 450/620 nm on a microplate ELISA reader. For each test sample, the titer was determined (S/P 100×) and the titer of ≥40 was considered positive for *C. burnetii* antibodies.

#### Leptospirosis

For the detection of anti-leptospiral antibodies, microscopic agglutination test (MAT) was employed using a panel of 18 reference *Leptospira* serovars covering 16 serogroups as described earlier [11]. For this purpose, 5–7-day-old cultures of various serovars grown in Ellinghausen–McCullough–Johnson–Harris medium (Difco™ *Leptospira* Medium Base and Enrichment EMJH, Bacton Dickinson, USA) at 28–30°C, at a concentration of 1–2 × 10^8^ leptospires/mL, were used in the MAT. To determine positive reactors (with titer ≥100 for one or more serovars) against different serogroups, a cutoff titer ≥100 was used as per the World Health Organization/World Organization for Animal Health recommendation for endemic settings. The test results of some of the serum samples for the anti-leptospiral and anti-*C. burnetii* antibodies status available in the laboratory records were used for analysis.

### Statistical analysis

The obtained qualitative data were subjected to statistical analysis (Chi-squared test) in Microsoft Office Excel 2016 to determine the association between *T. gondii* antibodies with a working null hypothesis (H0), independent (no association) of the presence of antibodies with the history of abortions and reproductive problems in cattle. The apparent prevalence was estimated as positive to the number of tested animals. Further, to know the relation of *T. gondii* antibodies, the odds (risk relation), risk of disease history (abortions and reproductive disorders), and its association with seropositivity were compared. Similarly, the presence of coexisting antibodies against *C. burnetii* and *Leptospira* spp. with toxoplasmosis was also analyzed.

## Results and Discussion

**Figure-1:**
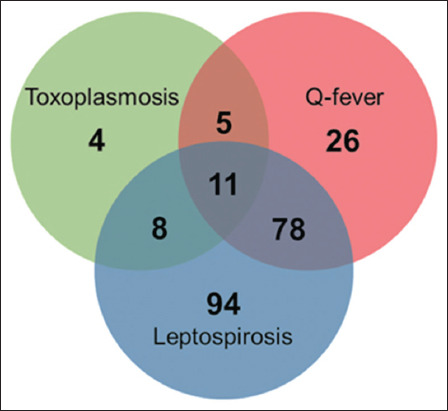
The past exposure coexisting antibodies against *Toxoplasma gondii, Coxiella burnetii*, and *Leptospira* spp. infection in the positive dairy cattle associated with reproductive disorders.

Of the 246 sera screened, 28 (11.4%) were found to be positive for anti-*T. gondii* antibodies in ELISA, of which 16 positive sera (16/28 = 57.1%) were associated with abortions, whereas 12 samples (12/28 = 42.9%) with other reproductive problems. The details of the serum samples screened for *T. gondii*, *Leptospira*, and *C. burnetii-*associated antibodies and their test results are summarized in Table-1. The observed high positivity of cattle against *Leptospira* and *C. burnetii* antibodies could be due to the samples being from suspected animals having reproductive problems of history [11, 12, 17]. The past exposure to coexisting antibodies of the positive cattle with reproductive disorders is depicted in Figure-1. Varying levels of prevalence (3%–76.3%) of *T. gondii* antibodies have been reported in many countries, including India [2, 5, 6, 18–24]. The seroprevalence of toxoplasmosis in cattle at different locations varies significantly within the country [4, 14]. The prevalence observed in this study (11%) is lower than the earlier seropositivity reports from South India (61.5%–71.8%) and 19.3% in North India [6, 25]. This might be due to the tested samples being from stall-fed or semi-stall-fed cattle dairy farms less exposed to infective cat feces [26]. However, it corroborates with the reported prevalence (ranging from 2.3% to 76%) in other countries. The prevalence differences between countries or regions might be due to the difference in sample size and techniques employed in different studies [4, 5, 18, 21]. The prevalence of anti-*C. burnetii* and *Leptospira* spp. antibodies at 50% and 78% levels, respectively, and 11% of *T. gondii* antibodies in cattle were observed, which has severe implications for both animal and public health as experienced by previous workers [16, 17]. The seropositivity was found to be 91.9% (226/246) for at least one of the screened zoonoses. This indicates the prevalence of antibodies against either of these pathogens in association with reproductive disorders (p < 0.005). Moreover, the proportion of cattle found to have *T. gondii* antibodies was only 1.8%, whereas 11.5% of *C. burnetii* antibodies and 41.6% of *Leptospira* spp. antibodies were detected.

Further, the predominantly mixed infections observed (Figure-1) were leptospirosis and Q fever (34.5%) followed by all three infections (4.87%); toxoplasmosis and leptospirosis (3.54%); and toxoplasmosis and Q fever (2.21%), which are corroborated with the reported data by various researchers in different studies. Adesiyun *et al*. [27] observed the seroprevalence of 38% and 33% for *C. burnetii* and *T. gondii*, respectively, while studying the epidemiology of the cattle for exposure to different abortifacient zoonotic pathogens. In contrast, Sekovanić *et al*. [28] reported a prevalence of 5% and 6% for Q fever and leptospirosis, respectively, without a single positive of brucellosis in cattle during 5 years of study in Croatia. The results revealed that the antibodies for at least one of the screened zoonoses were present and the other seronegative animals may be positive for either one or many other infectious abortifacients pathogens, as many pathogens have been reported to be associated with cattle reproductive problems [16].

The Chi-square test showed that seropositivity of toxoplasmosis (p < 0.02), Q fever (p < 0.001), and leptospirosis (p < 0.018) was significantly associated with reproductive problems in cattle and reproductive disorders in the farms. Further, the Chi-square test and risk relation analysis of the screened samples are available from the corresponding author upon a reasonable request. The previous studies [16, 17] have reported that these infections are responsible for reproductive failure and indicate that personnel working in slaughterhouses or meat handlers are at high risk, if proper hygiene is not maintained. The scavenging cats/felids in and around slaughterhouse premises or meat shop areas can pick up the infection by ingesting raw meat contaminated with *Toxoplasma* cysts and later become a source of infection. Hence, cattle carcasses in that area should be handled quite hygienically to break the life cycle. Similarly, the transmission of Q fever is mainly through the infected animals, their contaminated body fluids/secretion/aborted materials, contaminated aerosol particles or unpasteurized milk, and animal products; hence, the milk handlers are at high risk. For leptospirosis, young animals shed more bacteria in urine than older ones [29], causing health hazards to animal handlers.

On analysis, 15 out of 35 farms tested and were found to be positive for *T. gondii* antibodies (Table-1), invariably of at least one seropositive animal in each farm with none of the farms free from any of these infections and with no single farm associated alone with toxoplasmosis. There were 14 farms with all these infections; 18 farms were positive for Q fever and leptospirosis together; one farm with toxoplasmosis and Q fever, and two farms were positive only for leptospirosis. The seropositivity of toxoplasmosis farms showed the exposure of cattle and its circulation of *Toxoplasma* oocysts in the environment since vaccination is not being practiced. Further, the high seropositivity of Q fever along with leptospirosis might be due to exposure to introduced or healthy seropositive shedder [10, 11]. These results corroborate the reported data from other countries [5, 21, 22]. The seroprevalence of 19% with at least one seropositive cattle on 69% of the farms in Estonia was reported [15]. Further, a cattle-level prevalence of 13.3% associated with the reproductive disorder in 29 farms in Sudan [5] and a 3% prevalence of toxoplasmosis with 38% herd-level positivity in North Poland [20] were also reported.

This study has certain limitations, such as host factors were not available for multifactorial analysis; other infectious abortifacients agents causing reproductive disorders were not investigated. Further, cattle samples from each farm that did not have reproductive difficulties as a comparison control group are not available to establish the correlation. However, this is the first of its kind on the documentation of coexisting antibodies (past exposure with more than 1 of the pathogens was evident) against these abortifacient zoonotic pathogens in cattle with reproductive disorders in India. Although the study revealed serological evidence, it is not possible to determine the exact pathogen or its association with agents that possibly have potentialized the outcome of reproductive problems or if there was an association of agents.

## Conclusion

This study revealed the prevalence of *T. gondii* antibodies in dairy cattle associated with reproductive disorders with coexisting antibodies against *Leptospira* and *C. burnetii*. These seropositive cattle may act as a potential source of the infection by transmitting the disease to other animals and risk group personnel. These findings will be helpful in mitigating the disease burdens by implementing control measures. The serological studies, environmental sanitation measures, control of rodents and felids in close proximities to the premises, and the dissemination of epidemiological information about these zoonoses are relevant for better health of personnel on the farm.

## Data Availability

The datasets generated during this study are available from the corresponding author on a reasonable request.

## Authors’ Contributions

VB: Designed and conceptualized the experiment, interpreted the data, and drafted and revised the manuscript. AA and KVK: Carried out the experiments. KVK and GG: Interpreted the data and performed the statistical analysis. PPS: Provided diagnostics support. PPS and GG: Language editing of the manuscript. BRS: Provided guidance and support to conduct the research work. All authors have read and approved the final manuscript.
